# Same Name, Moving Target: Model Version Drift in Syntheses of Large Language Models for Mental Health

**DOI:** 10.2196/111307

**Published:** 2026-10-15

**Authors:** Bing Zhang Sr

**Affiliations:** 1 Tangshan People's Hospital, 65 Shengli Road, Tangshan, Hebei, 063000, China, 86 15383056090

**Keywords:** large language models, generative artificial intelligence, generative AI, GenAI, mental health, primary health care, screening, clinical decision support, psychotherapy, digital health, eHealth, PRISMA

I read with interest Leung et al’s [1] systematic review and meta-analysis of large language models (LLMs) in mental health care. The bivariate meta-analysis with Hartung-Knapp adjustments is methodologically sound, and the conclusion that the evidence is too heterogeneous, indirect, and uncertain to support routine unsupervised use is appropriately cautious. I wish to raise an unaddressed threat to the pooled estimates: model version drift.

Over the review’s search window (November 2022 to March 2026), “GPT-4” (OpenAI) did not denote a single intervention: released in March 2023, it was followed by API snapshots (gpt-4-0314, gpt-4-0613, gpt-4-1106-preview, and later GPT-4o releases) whose behavior differed materially. GPT-4’s accuracy on a prime-number identification task fell from 84% to 51% between the March and June 2023 snapshots, with parallel shifts on other fixed benchmarks including United States Medical Licensing Examination questions [2]. Version drift is thus a source of between-study heterogeneity that *I*² can quantify but not attribute.

The review’s data extraction captured only “model name, model type,” and the authors state that they “use the term ChatGPT without the version number when the original study authors did not report the version of GPT used” [1]. The authors rightly note that ChatGPT is an interface to different model versions; the corollary is that OpenAI made GPT-4o available in ChatGPT’s free tier in May 2024 [3], so studies reporting only “ChatGPT” may have evaluated different underlying systems and cannot be assigned to any model arm. Testing dates were neither extracted nor, in most primary studies, reported; the estimates pooled under GPT-4 (k=4) and GPT-3.5 (k=4) may each span several substantively different models. In effect, the pooled “GPT-4” estimate corresponds to no single system a clinician could deploy. This concern is distinct from the acknowledged limitation that few studies evaluated the newest model variants, which concerns currency rather than comparability.

The abstract transparently reports the full confidence and prediction intervals; my concern is narrower. Its comparative summary, that GPT-4 “had the higher pooled sensitivity,” appears without any qualification that model differences were not formally tested, since the arms were pooled separately. Given that version mixing may partly explain the apparent ordering, such a qualification would help readers avoid overreading the ranking.

The root cause lies in incomplete reporting by primary studies, which a review cannot recover. Publication dates, though, are always available and bound the plausible version set: a study published in September 2023 cannot have used gpt-4-1106-preview. I would welcome the authors’ assessment of whether (1) version and testing date could be reported for each pooled study (2), studies lacking a reported version could be examined in a sensitivity analysis, and (3) the abstract’s comparative sentence could note that differences were not formally tested. TRIPOD-LLM (Transparent Reporting of a Multivariable Prediction Model for Individual Prognosis Or Diagnosis–LLM) asks primary studies to identify models by name, version, and snapshot date (item 6a) [4]; syntheses should extract and report the same, clarifying the robustness of pooled estimates to version mixing.

## Abbreviations

LLM: large language model
TRIPOD-LLM: Transparent Reporting of a Multivariable Prediction Model for Individual Prognosis Or Diagnosis–Large Language Model
